# Pentoxifylline Enhances Antioxidative Capability and Promotes Mitochondrial Biogenesis in D-Galactose-Induced Aging Mice by Increasing Nrf2 and PGC-1*α* through the cAMP-CREB Pathway

**DOI:** 10.1155/2021/6695613

**Published:** 2021-06-22

**Authors:** Yu Wang, Tianyun Zhang, Hui Zhao, Chunxiao Qi, Xiaoming Ji, Hexin Yan, Rui Cui, Guoliang Zhang, Yunxiao Kang, Geming Shi

**Affiliations:** ^1^Department of Neurobiology, Hebei Medical University, Shijiazhuang 050017, China; ^2^Department of Anatomy, Hebei Medical University, Shijiazhuang 050017, China; ^3^Department of Histology and Embryology, Hebei University of Engineering, Handan 056002, China; ^4^Neuroscience Research Center, Hebei Medical University, Shijiazhuang 050017, China; ^5^Hebei Key Laboratory of Neurodegenerative Disease Mechanism, Hebei Medical University, Shijiazhuang 050017, China

## Abstract

Aging is a complex phenomenon associated with oxidative stress and mitochondrial dysfunction. The objective of this study was to investigate the potential ameliorative effects of the phosphodiesterase inhibitor pentoxifylline (PTX) on the aging process and its underlying mechanisms. We treated D-galactose- (D-gal-) induced aging mice with PTX and measured the changes in behavior, degree of oxidative damage, and mitochondrial ultrastructure and content as well as the expression of nuclear factor erythroid 2-related factor 2- (Nrf2-) mediated antioxidant genes and peroxisome proliferator-activated receptor-gamma coactivator 1-alpha- (PGC-1*α*-) dependent mitochondrial biogenesis genes. The results demonstrated that PTX improved cognitive deficits, reduced oxidative damage, ameliorated abnormal mitochondrial ultrastructure, increased mitochondrial content and Nrf2 activation, and upregulated antioxidant and mitochondrial biogenesis gene expression in the hippocampus of wild-type aging mice. However, the above antiaging effects of PTX were obviously decreased in the brains of Nrf2-deficient D-gal-induced aging mice. Moreover, in hydrogen peroxide-treated SH-SY5Y cells, we found that cyclic adenosine monophosphate (cAMP) response element-binding protein (CREB) and Nrf2/PGC-1*α* act in a linear way by CREB siRNA transfection. Thus, PTX administration improved the aging-related decline in brain function by enhancing antioxidative capability and promoting mitochondrial biogenesis, which might depend on increasing Nrf2 and PGC-1*α* by activating the cAMP-CREB pathway.

## 1. Introduction

Aging is a major factor underlying a gradual decline in brain function and has been characterized by pathological features such as reactive oxygen species- (ROS-) induced oxidative stress and mitochondrial dysfunction [1–4]. Mitochondria are the primary source and target of ROS, and they generate ROS as a natural byproduct of oxidative phosphorylation and scavenge ROS by efficient antioxidant defense systems. The endogenous antioxidant capacity gradually decreases with aging, which leads to massive accumulations of oxidative damage to proteins, nucleic acids, and lipids [5]. In addition, oxidative stress may induce oxidative damage to mitochondria, which need to promote mitochondrial biogenesis to maintain normal mitochondrial function. Previous studies have revealed that a decrease in mitochondrial biogenesis during aging reduces the turnover of mitochondrial components, which further weakens and impairs mitochondrial function and antioxidant defense systems [6–8]. Ultimately, the above pathological changes potentially accelerate aging and the development of age-associated neurodegenerative diseases [1, 9, 10]. Hence, enhancing antioxidative capability and promoting mitochondrial biogenesis may represent a rational strategy for maintaining normal brain function in aged organisms.

Nuclear factor-erythroid 2-related factor 2 (Nrf2), the most critical transcription factor in antioxidant defense systems, coordinates the expression of detoxification and antioxidant genes by binding to antioxidant response elements (ARE), such as heme oxygenase 1 (HO-1), NAD(P)H quinone oxidoreductase 1 (NQO1), superoxide dismutase (SOD), catalase (CAT), and glutathione peroxidase (GPx) [11–13]. Activated Nrf2 also directly or indirectly upregulates the expression of mitochondrial biogenesis-related genes, such as peroxisome proliferator-activated receptor-gamma coactivator 1-alpha (PGC-1*α*), nuclear respiratory factor 1 (NRF-1), and mitochondrial transcription factor A (TFAM) [14–18]. Various scientific studies have demonstrated that the expression of Nrf2 progressively decreases with age and that Nrf2 activation might be a potential therapeutic target to delay the aging and aging-related neurodegenerative process [12, 13, 19, 20].

Pentoxifylline (PTX), a methylxanthine derivative, has been applied clinically for the treatment of peripheral vascular diseases and cerebrovascular diseases [21]. Furthermore, it was reported that PTX has ameliorative effects on aging or other pathological processes, such as improving behavioral deficits and anti-inflammatory activity, restoring dopaminergic neurochemical levels and antioxidant activity, and ameliorating mitochondrial function [22–25]. These findings are also supported by our earlier research on the antiaging effects of PTX in aged rats, in which we found that the beneficial function might be related to Nrf2 activation [22]. However, the precise molecular mechanisms of PTX-induced neuroprotective effects on the aging brain are unknown.

PTX is a nonspecific phosphodiesterase (PDE) inhibitor that can modulate the intracellular cyclic adenosine monophosphate (cAMP) signaling pathway [26]. The cAMP-cAMP response element-binding protein (CREB) signaling pathway is associated with synapse formation, memory enhancement [27, 28], ROS detoxification [29], and mitochondrial biogenesis [30, 31]. A previous study indicated that potential transcriptional cooperation may occur between CREB and Nrf2 to induce HO-1 expression and enhance apoptosis resistance [32]. According to the above findings, we hypothesized that PTX might potentiate the function of antioxidant defense systems and promote mitochondrial biogenesis by activating the cAMP-CREB and Nrf2-ARE pathways.

Large amounts of evidence have confirmed that injection of D-galactose (D-gal) could accelerate the aging process in rodents by increasing ROS formation and the accumulation of advanced glycation end products (AGEs) [33–36]. The above pathological changes further induce oxidative damage and mitochondrial dysfunction in the brains of experimental animals and lead to cognitive deficits [37–39]. Therefore, we established D-gal-induced aging models in wild-type and Nrf2-deficient mice and a hydrogen peroxide- (H_2_O_2_-) induced SH-SY5Y cell model to investigate the potential molecular mechanisms underlying PTX-induced antiaging effects. We found that PTX administration improved cognitive deficits, enhanced antioxidative capability, and promoted mitochondrial biogenesis in D-gal-induced aging mice, which might be closely related to the upregulation of Nrf2 and PGC-1*α* through the cAMP-CREB pathway.

## 2. Materials and Methods

### 2.1. Animals and Housing

Healthy male wild-type and Nrf2-deficient CD1/ICR mice were obtained from Professor C. Li (Department of Neurology, Second Hospital of Hebei Medical University, Shijiazhuang, China) and genotyped using the following primers: *NRF5* (in the Nrf2 gene), 5′-TGGACGGGACTATTGAAGGCTG-3′; *NAS* (in the Nrf2 gene), 5′-GCCGCCTTTTCAGTAGATGGAGG-3′; and *NLACZ* (in the LacZ gene), 5′-GCGGATTGACCGTAATGGGATAGG-3′. Male wild-type and Nrf2-deficient CD1/ICR mice (2-3 months old, weighing 29 ± 3 g) were provided food and water *ad libitum* and housed under controlled conditions of 22 ± 2°C, 50-60% relative humidity, and a 12-hour light-dark diurnal cycle (lights on at 6 : 00 AM). The experimental procedures adhered to the “Guidelines for the Care and Use of Mammals in Neuroscience and Behavioral Research” and were approved by the Committee of Ethics on Animal Experiments at Hebei Medical University.

### 2.2. Experiment 1

**Figure 1:**
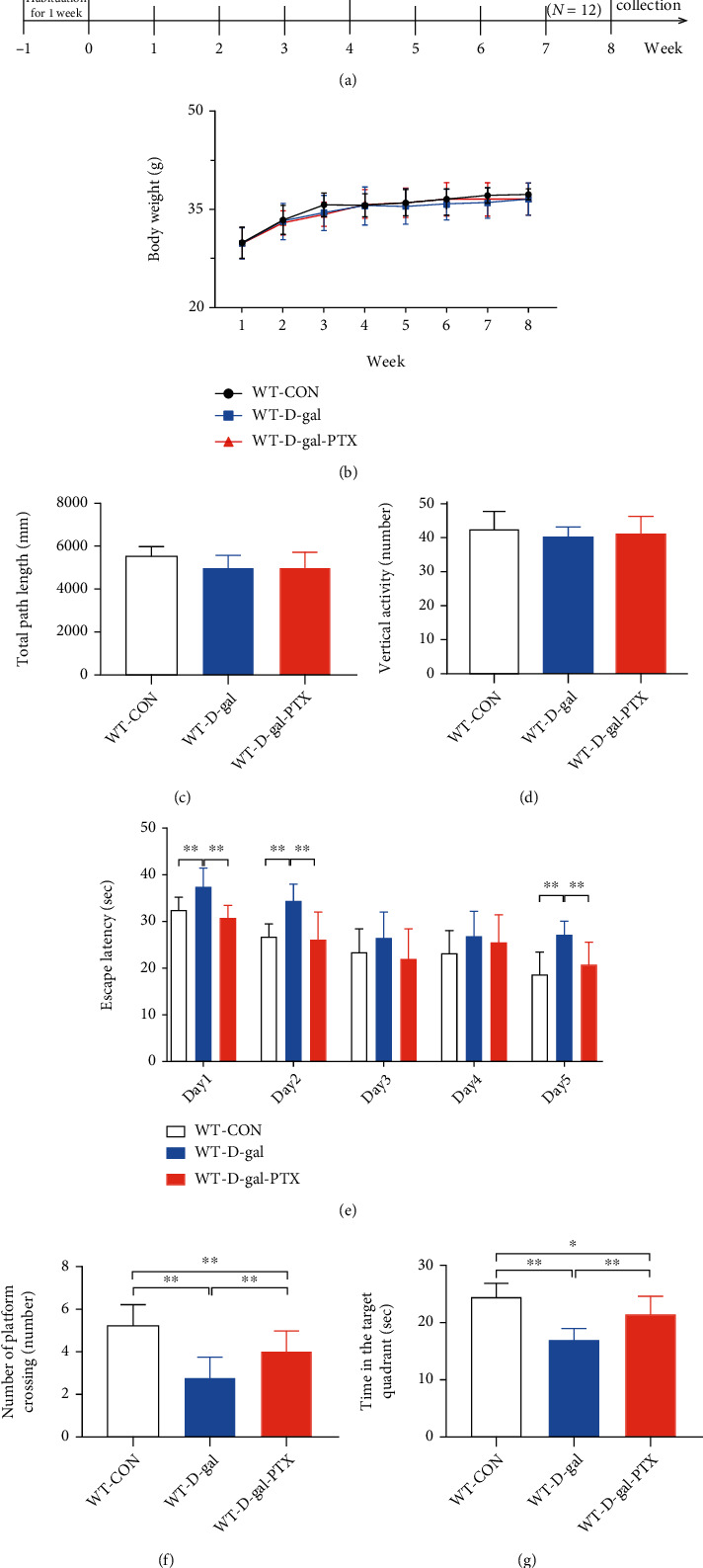
Effects of PTX administration on the body weight and behavioral deficits of D-gal-induced aging mice. (a) Illustration of the research process, including D-gal administration, PTX treatment, and behavioral tests. Effects of PTX administration on (b) body weight. Effects of PTX administration on behavioral parameters, including the (c) total path length, (d) vertical activity, (e) escape latency, (f) number of platform crossings, and (g) time in the target quadrant. i.h.: hypodermic injection; i.p.: intraperitoneal injection; OF: open-field test; MWM: Morris water maze test. Data are expressed as the mean ± S.D. (*n* = 12 mice/group). ^∗^*P* < 0.05 and ^∗∗^*P* < 0.01.

After one week of adaptive feeding, eighty-one wild-type mice were randomly divided into three groups: wild-type normal control group (WT-CON), wild-type D-gal model group (WT-D-gal), and wild-type D-gal and PTX treatment group (WT-D-gal-PTX). D-gal or PTX was dissolved in 0.9% normal saline, and the dose of D-gal or PTX was administered to mice based on previous studies [22, 34, 37, 40]. Mice were administered normal saline once daily in the WT-CON group and subjected to D-gal (TCI, 100 mg/kg/d) by hypodermic injection for 8 weeks in the other groups. Mice in the WT-D-gal-PTX group were treated with PTX (Sigma, 60 mg/kg/d) by intraperitoneal injection 1 h before the D-gal treatment for the last four weeks. After administration, the effects of PTX treatment on D-gal-induced aging in all the mice were investigated by analyzing the changes in behavior, oxidative balance status parameters, mitochondrial ultrastructure, mitochondrial content, and antioxidant and mitochondrial biogenesis-related gene expression (Figure 1(a)).

### 2.3. Experiment 2

Eighty-one Nrf2-deficient mice were used to investigate the effects of Nrf2 activation on the PTX-induced antiaging process. After acclimating for one week, the mice were randomly divided into three groups: Nrf2-deficient normal control group (KO-CON), Nrf2-deficient D-gal model group (KO-D-gal), and Nrf2-deficient D-gal and PTX treatment group (KO-D-gal-PTX). The three groups were subjected to the same treatment as in experiment 1. In addition, data from the WT-CON group were added to experiment 2 to analyze the similarities and differences between the wild-type and Nrf2-deficient mice.

### 2.4. Open Field Test

In the last week of experiment 1, the mice in the three groups were subjected to the open-field test. The open-field apparatus was a black square arena (50 cm in diameter and 35 cm in height) illuminated with 20 lux light. Tests were performed in a sound-attenuating chamber from 8 : 00 AM to 3 : 00 PM. Each mouse in the three groups was individually placed at the center of the arena and recorded with a ZH-ZFT open-field experimental video analysis system (Zhenghua, Anhui, China) for 5 min according to a previously described procedure with slight modifications [41]. Two behavioral parameters were observed in the study, namely, total path length (the distance of mice walking around) and vertical activity (the number of mice standing up on hind feet). After each test, 70% ethanol was used to remove the mouse odor from the apparatus.

### 2.5. Morris Water Maze Test

The mice in experiment 1 were tested for spatial learning and memory ability in the last six days of PTX treatment. The water maze was located in a quiet room and included a circular water tank (120 cm in diameter, 40 cm high) that was partially filled with water (22 ± 2°C) to a depth of 15.5 cm, and it was rendered opaque by adding ink. The pool was divided virtually into four equal quadrants. A black platform (9 cm in diameter, 14.5 cm in height) was hidden 1 cm below the surface of the water in a fixed location. The maze was surrounded by external visual cues to aid in the spatial orientation of the mice. The Morris water maze tasks consisted of training trials and spatial probe trials. The text was carried out as previously described with slight modifications [41]. The escape latency, number of platform crossings, and time in the target quadrant were documented for further analysis. All data were recorded with a computerized video system (Zhishuduobao, Beijing, China).

### 2.6. Sample Preparation

At the end of the two experiments, mice were sacrificed and the brains were removed quickly. Bilateral hippocampi were dissected on an ice-cold plate using a scalpel under a stereomicroscope. The hippocampi of the mice in each group were frozen in liquid nitrogen and stored at −80°C until further analysis, including the malondialdehyde (MDA) levels, protein carbonyl (PC) content, citrate synthase (CS) activity, mtDNA copy number, real-time quantitative PCR (qPCR), and Western blot based on the study purposes. Nine mice in each group were anaesthetized by intraperitoneal injection of 1% pentobarbital sodium (50 mg/kg). Six mice from each group were perfused transcardially with 4% paraformaldehyde (in 0.1 M phosphate buffer, pH 7.4) for later hematoxylin-eosin (HE) staining or immunohistochemistry (IHC) analysis. The remaining three mice of each group were perfused transcardially with fixative (2% paraformaldehyde and 1.25% glutaraldehyde) for mitochondrial ultrastructure analysis.

### 2.7. Histological Analysis of the Hippocampus

Six brains from each group were postfixed in the same fixative for 4 h (4°C), dehydrated in graded ethanol, cleared in xylene, and embedded in paraffin wax. Paraffin-embedded brain blocks were sliced into 5 *μ*m coronal sections. After deparaffinization and hydration, the sections containing the hippocampus were processed for HE staining or IHC analysis. For the analysis of 3-nitrotyrosine (3-NT), a biomarker of protein nitration, sections from experimental mice were subjected to antigen retrieval (in 0.01 M citrate buffer, pH 6.0) in a pressure cooker for 5 min. The brain sections were incubated with 5% normal goat serum to block nonspecific binding, which was followed by an overnight incubation with mouse anti-3-nitrotyrosine (39B6) antibody (1 : 100, Santa Cruz Biotechnology) at 4°C. After washing, the sections were immersed in 0.3% H_2_O_2_ in methanol for 30 min to abolish endogenous peroxidase activity. After three washes in phosphate buffer, the sections were incubated with goat anti-mouse IgM/HRP (1 : 500, Sera Care) for 2 h at room temperature (RT). All sections were stained for 5 min in 0.05 M Tris-HCl buffer (containing 0.05% diaminobenzidine and 0.03% H_2_O_2_, pH 7.6) to visualize 3-NT-positive cells. The average optical density (AOD) of 3-NT immunoreactive (3-NT-IR) intensity and the percentage of HE-stained cells with karyopycnosis in the hippocampal CA1, CA3, and dentate gyrus (DG) regions were measured by Image-Pro Plus 6.0 (Media Cybernetics, USA).

### 2.8. Mitochondrial Ultrastructure Analysis of the Hippocampus

The hippocampi of each group were dissected and fixed in fixative for 2 h. After three washes in phosphate buffer, the tissues were postfixed with 1% osmium tetroxide for 2 h, dehydrated in acetone, and embedded in araldite. Ultrathin sections (70 nm) were obtained with a microtome (UC-7, Leica, Austria). After staining with uranyl acetate (10 min) and lead citrate (5 min), the sections were examined by a transmission electron microscope (Hitachi HT7800, Japan) operated at 80 kV. For the electron microscopy (EM) image analyses, individual mitochondria were manually outlined, the mitochondrial number and size were measured using Image-Pro Plus 6.0 (Media Cybernetics, USA) at ×3000 magnification, and the mitochondrial ultrastructure was analyzed at ×25000 magnification.

### 2.9. MDA Detection

MDA is the product of lipid peroxidation and a biomarker of ROS-mediated cell membrane damage. The unilateral hippocampal tissue blocks in each group were weighed and homogenized with 10% (w/v) ice-cold 0.1 M phosphate buffer (pH 7.4). The supernatant from centrifuged homogenates was used to assess MDA levels using a detection kit following the manufacturer's instructions (A003-2-2, Nanjing Jiancheng Bioengineering Institute, China).

### 2.10. PC Assay

PC, a biomarker of protein oxidation, was measured as previously described [42]. Briefly, the tissue blocks in each group were homogenized with 10% (w/v) ice-cold extracting solution and centrifuged at 8,000 rpm for 10 min (4°C). The supernatants were collected and subjected to a biochemical evaluation according to the protocol of the Protein Carbonyl Content Determination Kit (G0130W, Suzhou Geruisi Biotechnology Institute, China). The PC content was expressed as *μ*mol/g protein.

### 2.11. CS Activity Assay

Mitochondria from hippocampal tissue blocks were isolated using the Minute™ Mitochondria Isolation kit (cat. no. MP-007, Invent Biotechnologies, USA). Subsequently, isolated mitochondria were dissolved in Minute™ Non-Denatured Protein Solubilization Reagent (cat. no. WA-010, Invent Biotechnologies, USA) and used to evaluate CS activity based on spectrophotometry at 412 nm following the instructions of the MitoCheck® Citrate Synthase Activity Assay Kit (Item No. 701040, Cayman, USA).

### 2.12. mtDNA Copy Number Determination

Hippocampal genomic DNA was extracted using an Animal Tissue Genomic DNA Kit (ZP307-2, Beijing Zoman Biotechnology Institute, China). The mtDNA copy number was normalized to the single-copy nuclear *HK2* gene and measured via qPCR analysis and the 2^-*ΔΔ*Ct^ method. Primer sequences for the mitochondrial segment were as follows: 5′-CCGCAAGGGAAAGATGAAAGAC-3′ and 5′-TCGTTTGGTTTCGGGGTTTC-3′. Primer sequences for the nuclear control were as follows: 5′-GCCAGCCTCTCCTGATTTTAGTGT-3′ and 5′-GGGAACACAAAAGACCTCTTCTGG-3′. Accession numbers of the genes for primers are listed in Table S1.

### 2.13. Cell Culture

Human neuroblastoma SH-SY5Y cells are widely used in *in vitro* studies, such as proliferation, apoptosis, and H_2_O_2_-induced oxidative damage. SH-SY5Y cells (ATCC, Manassas, VA) were maintained in DMEM/F12 (Biological Industries) containing 10% fetal bovine serum (FBS) (Biological Industries) and 1% penicillin and streptomycin at 37°C (5% CO_2_/95% air). SH-SY5Y cells (2.5 × 10^3^ pfu/well) were seeded into a 96-well plate to detect cell viability after different treatments by 3-(4,5-dimethylthiazol-2-yl)-2,5-diphenyltetrazolium bromide (MTT) assay. After reaching 70% confluence, the cells were treated with varying concentrations of PTX (0, 0.25, 0.5, 1, 2, or 4 mM) for a period of 2 h to determine the maximum nontoxic dose or with different concentrations of H_2_O_2_ (0, 50, 100, 200, 400, or 800 *μ*M) for a period of 24 h to determine the optimal concentration of H_2_O_2_. At the end of treatment, the culture medium was replaced with medium containing MTT at a final concentration of 0.5 mg/ml, and the cells were incubated at 37°C. After 4 h, the culture supernatant was carefully removed and purple formazan crystals were dissolved by adding 150 *μ*l/well dimethyl sulfoxide for 10 min. The absorbance was determined at 570 nm.

To investigate the protective effects of PTX on H_2_O_2_-induced oxidative stress in SH-SY5Y cells, the cells were assigned to six groups (control, H_2_O_2_, 0.25 mM PTX+H_2_O_2_, 0.5 mM PTX+H_2_O_2_, 1 mM PTX+H_2_O_2_, and 2 mM PTX+H_2_O_2_). After 2 h of PTX pretreatment, the cells were exposed to the optimal concentration of H_2_O_2_ (200 *μ*M was predetermined in this study) for another 24 h and then subjected to cell viability and Western blot analyses.

### 2.14. Transient Transfection with siRNA Targeting CREB

Small interfering RNA (siRNA) oligonucleotides targeting human CREB (siCREB-1: 5′-GCTCGAGAGTGTCGTAGAA-3′; siCREB-2: 5′-GAGTCAGTGGATAGTGTAA-3′; siCREB-3: 5′-CAACCAAGTTGTTGTTCAA-3′) and nonspecific oligonucleotides (siRNA-negative control, NC) were ordered from RiboBio (Suzhou, China). After reaching 50-60% confluence, the SH-SY5Y cells were transiently transfected with siRNA using HighGene transfection reagent (RM09014, ABclonal, China). The efficiency of CREB gene silencing was determined at 48 h posttransfection via qPCR and Western blot analyses.

To investigate the relationship between the CREB and Nrf2/PGC-1*α* pathways, we assigned the SH-SY5Y cells to six groups (NC, NC-H_2_O_2_, NC-H_2_O_2_-PTX, siCREB, siCREB-H_2_O_2_, and siCREB-H_2_O_2_-PTX). The cells were first infected with siCREB (MOI = 50, CREB silencing efficiency > 70%) or NC as appropriate. Afterwards, the infected cells were maintained for 24 h in fresh complete medium and then treated with 1 mM PTX for 2 h and 200 *μ*M H_2_O_2_ for 24 h. This PTX dose was chosen based on the results of the above experiments. After treatment, the cells were subjected to Western blot analysis.

### 2.15. qPCR Analysis

Total RNA was extracted with TRIzol (Invitrogen) for reverse transcription. cDNA was synthesized from total RNA (1 *μ*g) using a first-strand cDNA synthesis kit (RK20402, ABclonal). SYBR green qPCR mix (ZS-M-1009, Beijing Zoman Biotechnology Institute, China) was used according to the manufacturer's instructions. Then, the PCR products were analyzed by melting curve analysis to confirm the specificity of amplification. The expression of genes associated with senescence, antioxidant, and mitochondrial biogenesis was detected, and relative quantification was performed using the 2^−*ΔΔ*Ct^ method. *GAPDH* was used as a reference gene in all calculations. The sets of primers are listed in Table 1, and accession numbers of the genes for primers are listed in Table S1.

### 2.16. Western Blot Analysis

The proteins from hippocampal tissue blocks or SH-SY5Y cells were homogenized in ice-cold radioimmunoprecipitation assay (RIPA) buffer (cat. no. R0010, Solarbio, China) containing 1% phenylmethanesulfonyl fluoride. An immunoblotting analysis was performed following a previous method [43]. The presence of particular proteins was examined using rabbit anti-p-CREB (Ser133) antibody (1 : 500, Huabio), rabbit anti-CREB antibody (1 : 1000, Huabio), rabbit anti-p-Nrf2 (Ser40) antibody (1 : 300, Affinity), mouse anti-Nrf2 antibody (1 : 500, Abcam), rabbit anti-HO-1 antibody (1 : 300, Affinity), mouse anti-NQO1 antibody (1 : 300, Abcam), rabbit anti-PGC-1*α* antibody (1 : 300, ABclonal), anti-NRF-1 antibody (1 : 1000, ABclonal), rabbit anti-TFAM antibody (1 : 500, GeneTex), rabbit anti-*β*-actin antibody (1 : 10000, Santa Cruz Biotechnology), or rabbit anti-GAPDH antibody (1 : 1000, Abcam). After washing three times, the membrane was incubated for 2 h in IRDye® 800-conjugated goat anti-rabbit secondary antibody (1 : 10000, Rockland) or goat anti-mouse secondary antibody (1 : 10000, Rockland) at RT. The relative band densities were measured by an Odyssey infrared scanner (LI-COR Biosciences, USA). The densitometry values of the studied target proteins were normalized relative to that of reference proteins (*β*-actin or GAPDH) or nonphosphorylated related proteins.

### 2.17. Statistical Analysis

Data are described using the mean ± standard deviation (S.D.). Grubb's test was applied to remove possible outliers. Tests of normality and homogeneity of variance were applied to all of the data. If both a normal distribution (*P* > 0.1) and homogeneity of variance (*P* > 0.05) were found, then a parametric test was performed by one-way analysis of variance (one-way ANOVA) followed by Tukey's honestly significant difference (Tukey's HSD) post hoc test for multiple comparisons. Otherwise, nonparametric statistical tests were performed by the Games-Howell procedure for post hoc analysis between groups [44]. Statistical analysis was performed using the Statistical Package for the Social Sciences 21 software (SPSS Inc., Chicago, IL, USA) and Prism 8.0 (GraphPad Software Inc., La Jolla, CA). Differences were considered statistically significant at a *P* value of less than 0.05.

## 3. Results

### 3.1. Effects of PTX Administration on Body Weight and Spontaneous Activity in D-Gal-Induced Aging Mice

During experiment 1, we monitored the body weight changes of experimental mice for 8 weeks (Figure 1(b)). In the last week, we performed an open-field test to observe changes in spontaneous activity among the WT-CON, WT-D-gal, and WT-D-gal-PTX groups. The total path length (Figure 1(c)) and vertical activity (Figure 1(d)) were measured to assess the motor ability of experimental animals. Significant differences were not observed in the body weight, total path length, or vertical activity among the three groups, indicating that D-gal and PTX administration had little effect on the body weight and spontaneous activity in experimental mice.

### 3.2. PTX Administration Improved Cognitive Function in D-Gal-Induced Aging Mice

Next, to evaluate the effects of PTX on spatial learning and memory capability in aging mice, experimental animals were trained and tested using a water maze test. Group differences in escape latency (Figure 1(e), day 1, day 2, and day 5: *P* < 0.01), number of platform crossings (Figure 1(f), *P* < 0.01), and time in the target quadrant (Figure 1(g), *P* < 0.01) were found among the three groups. The escape latency in the WT-D-gal group was markedly more prolonged than that in the WT-CON group (day 1, day 2, and day 5: *P* < 0.01). In addition, the number of platform crossings was decreased (*P* < 0.01) and the time spent in the target quadrant was shortened (*P* < 0.01) in the WT-D-gal group compared to the WT-CON group. The findings indicated that D-gal induced cognitive deficits in experimental mice. However, the administration of PTX shortened the escape latency to reach the platform (day 1, day 2, and day 5: *P* < 0.01), increased the number of platform crossings (*P* < 0.01), and extended the time spent in the target quadrant (*P* < 0.01) in the WT-D-gal-PTX group compared to the WT-D-gal group. These results suggested that PTX could effectively improve cognitive deficits in D-gal-induced aging mice.

### 3.3. PTX Administration Alleviated Oxidative Damage in the Brains of D-Gal-Induced Aging Mice

Oxidative damage in the hippocampus is one of the most crucial mechanisms underlying D-gal-induced cognitive deficits and may cause neuronal loss and morphological changes. Thus, hippocampal neurons in the CA1, CA3, and DG regions were chosen to observe karyopycnosis by HE staining. Moreover, the 3-NT-IR, PC content, and MDA levels, which are three critical parameters of oxidative stress, were measured concurrently to detect the effects of PTX treatment on oxidative damage in the brains of D-gal-induced aging mice.

**Figure 2:**
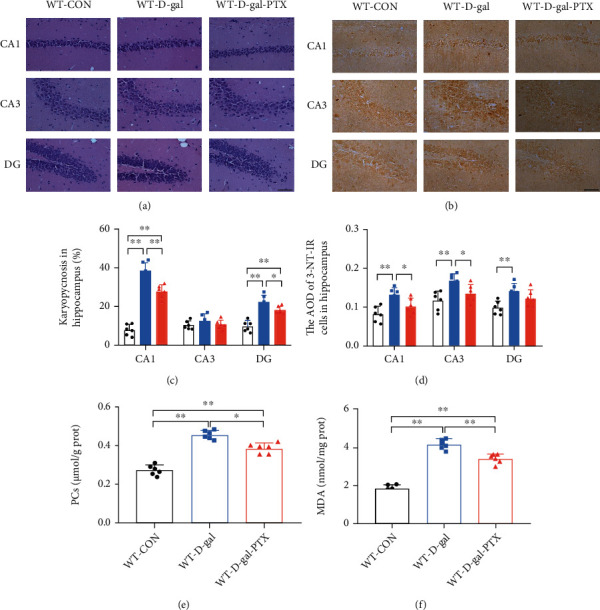
Effects of PTX administration on oxidative damage in the hippocampus of D-gal-induced aging mice as determined by (a, c) HE staining for karyopycnosis, (b, d) IHC staining for 3-NT, (e) PC assay, and (f) MDA detection. Scale bars = 50 *μ*m. Data are expressed as the mean ± S.D. (*n* = 6 mice/group). ^∗^*P* < 0.05 and ^∗∗^*P* < 0.01.

Group differences among the three groups were found in the percentage of karyopycnosis (Figures 2(a) and 2(c), CA1 and DG regions, *P* < 0.01), the AOD of 3-NT-IR cells (Figures 2(b) and 2(d), *P* < 0.01), PC content (Figures 2(e), *P* < 0.01), and MDA levels (Figure 2(f), *P* < 0.01) in the hippocampus. Post hoc tests revealed that the percentage of karyopycnosis (except for the CA3 region), the AOD of 3-NT-IR cells, and the levels of PCs and MDA were significantly elevated in the WT-D-gal group compared to the WT-CON group (*P* < 0.01). Meanwhile, except for the karyopycnosis in the CA3 region and the 3-NT-IR AOD in the DG region, the above four parameters were decreased in the WT-D-gal-PTX group compared to the WT-D-gal group (karyopycnosis in the CA1 region and MDA levels: *P* < 0.01 and others: *P* < 0.05). PTX administration alleviated oxidative damage in the brains of D-gal-induced aging mice.

### 3.4. PTX Administration Promoted Mitochondrial Biogenesis in the Brains of D-Gal-Induced Aging Mice

**Figure 3:**
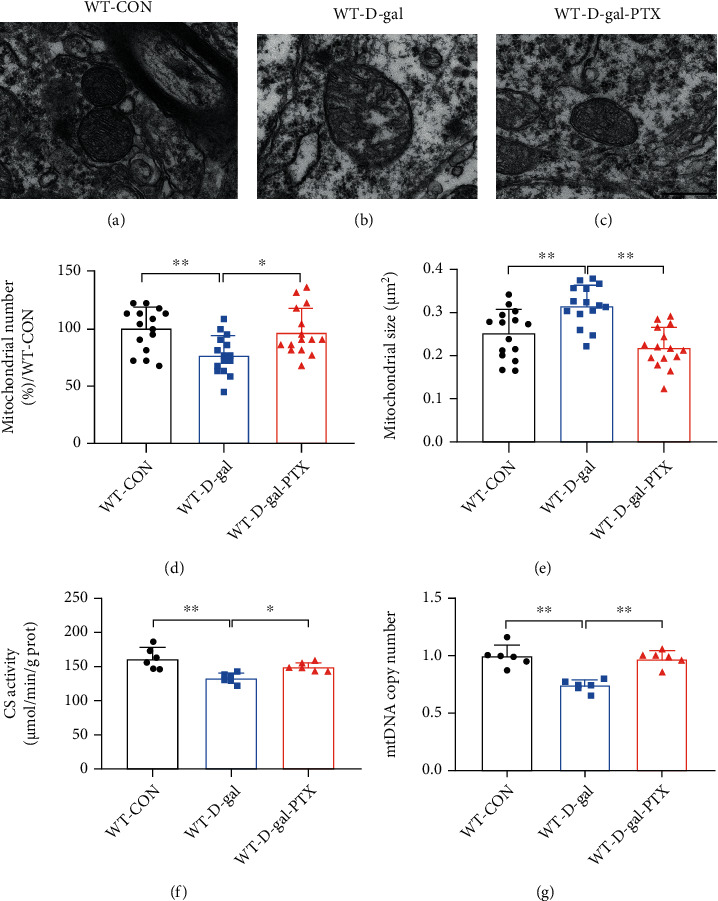
Effects of PTX administration on mitochondrial biogenesis in the hippocampus of D-gal-induced aging mice. Using EM, mitochondrial ultrastructure images were collected from the (a) WT-CON, (b) WT-D-gal, and (c) WT-D-gal-PTX groups. Scale bars = 500 nm. Quantitative morphometric measurements of the average level of (d) mitochondrial number and (e) mitochondrial size based on an analysis of 5 images per sample from each group (*n* = 3 mice/group). (f) CS activity and (g) mtDNA copy number were tested to measure mitochondrial content among the three groups (*n* = 6 mice/group). Data are expressed as the mean ± S.D.^∗^*P* < 0.05 and ^∗∗^*P* < 0.01.

With age, mitochondria deteriorate, the structure, and mtDNA show accumulating damage, and the activity of mitochondrial enzymes (e.g., CS) gradually decreases, and these changes lead to a reduction in mitochondrial content and biogenesis [10]. To examine the potential effects of PTX on mitochondrial biogenesis, we analyzed mitochondrial morphology and content in the hippocampus of D-gal-induced aging mice. There were striking differences in the mitochondrial ultrastructure (Figures 3(a)–3(c)), number (Figure 3(d), *P* < 0.01), and size (Figure 3(e), *P* < 0.01) among the three experimental groups. The WT-CON group maintained a normal structure and size of mitochondria, which presented with thin and uniform cristae and surrounded by clear inner and outer membranes. However, most mitochondria from the WT-D-gal group showed various patterns of structural abnormalities, including swollen mitochondria, disorganized cristae, and ruptured inner and outer membranes. After the PTX treatment, the above abnormal ultrastructural alterations in mitochondria were improved. In addition, EM analyses showed that the mitochondrial number was reduced in the WT-D-gal group (*P* < 0.01) but elevated in the WT-D-gal-PTX group (*P* < 0.05). Meanwhile, the mitochondrial volume was increased in the WT-D-gal group (*P* < 0.01) and decreased in the WT-D-gal-PTX group (*P* < 0.01). Consistent with the EM data, the CS activity (Figure 3(f)) and mtDNA copy number (Figure 3(g)) were reduced by 17.63% (*P* < 0.01) and 25.71% (*P* < 0.01) in the WT-D-gal group compared to the WT-CON group, respectively, and increased by 12.00% (*P* < 0.05) and 31.90% (*P* < 0.01) in the WT-D-gal-PTX group relative to the WT-D-gal group, respectively. The results indicated that PTX administration promoted mitochondrial biogenesis in the brains of D-gal-induced aging mice.

### 3.5. PTX Administration Upregulated Antioxidant and Mitochondrial Biogenesis-Related Gene Expression in the Brains of D-Gal-Induced Aging Mice

**Figure 4:**
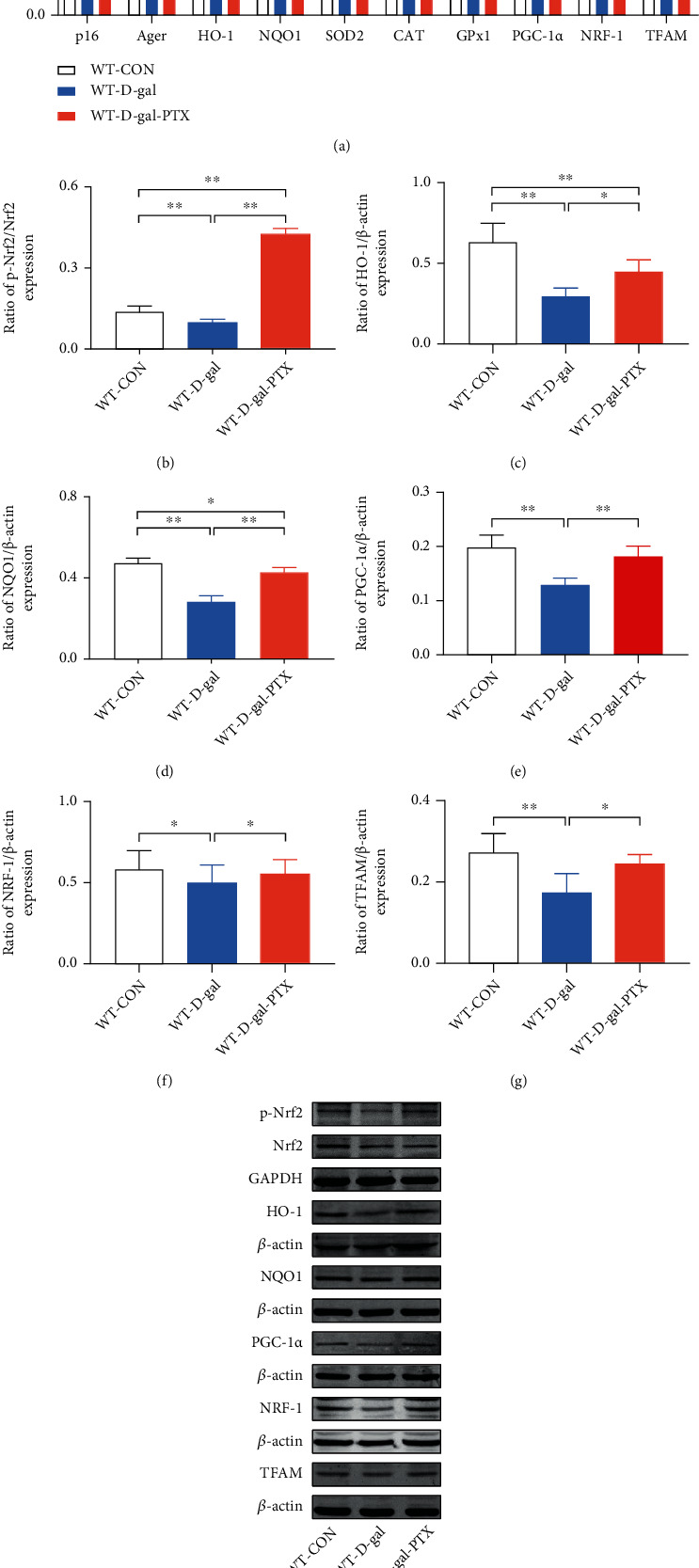
Effects of PTX administration on the expression of antioxidant and mitochondrial biogenesis-related genes in the hippocampus of D-gal-induced aging mice. *p16*, *Ager*, *HO-1*, *NQO1*, *SOD2*, *CAT*, *GPx1*, *PGC-1α*, *NRF-1*, and *TFAM* mRNA levels were detected by qPCR. (a) *GAPDH* was used as an internal control. Densitometry analysis of (b) p-Nrf2/Nrf2, (c) HO-1/*β*-actin, (d) NQO1/*β*-actin, (e) PGC-1*α*/*β*-actin, (f) NRF-1/*β*-actin, and (g) TFAM/*β*-actin. (h) Representative Western blots of the related protein levels mentioned above. Data are expressed as the mean ± S.D. (*n* = 6 mice/group). ^∗^*P* < 0.05 and ^∗∗^*P* < 0.01.

Based on the mechanisms of D-gal-induced aging, we further analyzed the expression of the senescence-associated gene *p16* and *Ager* (AGE receptors), which are two aging markers [45, 46]. Moreover, according to the roles of Nrf2 in antioxidant defense systems and the altered oxidative balance status in PTX-treated aging mice, the effects of PTX administration on Nrf2, HO-1, NQO1, SOD2, CAT, and GPx1 gene expression were analyzed in D-gal-induced aging mouse brains. Group differences in *p16*, *Ager*, *HO-1*, *NQO1*, *SOD2*, *CAT*, and *GPx1* mRNA levels (Figure 4(a), *P* < 0.01) as well as p-Nrf2, HO-1, and NQO1 protein levels (Figures 4(b)–4(d) and 4(h), *P* < 0.01) were found among the three groups. As shown in Figure 4, the results in the WT-D-gal group indicated that D-gal increased *p16* and *Ager* mRNA levels, decreased *HO-1*, *NQO1*, *SOD2*, *CAT*, and *GPx1* mRNA levels, and downregulated p-Nrf2, HO-1, and NQO1 protein levels in the hippocampus compared to the WT-CON group (*P* < 0.01). However, PTX administration was able to significantly reverse these deficits (mRNA levels: *GPx1*: not significant and others: *P* < 0.01; protein levels: HO-1: *P* < 0.05 and p-Nrf2 and NQO1: *P* < 0.01). The elevated p-Nrf2 levels in PTX-treated aging mice indicated the activation of Nrf2. These findings suggested that PTX administration attenuated D-gal-induced aging and enhanced antioxidative capability in aging mice.

In view of the modulation of mitochondrial biogenesis by Nrf2, we measured PGC-1*α*, NRF-1, and TFAM expression in the brains of D-gal-induced aging mice. Differences in the expression of PGC-1*α*, NRF-1, and TFAM at the mRNA and protein levels were observed among the experimental groups (Figures 4(a) and 4(e)–4(h), *P* < 0.01). The results revealed that the expression of PGC-1*α*, NRF-1, and TFAM at the mRNA and protein levels was significantly downregulated in the WT-D-gal group compared with the WT-CON group (mRNA levels: *P* < 0.01; protein levels: NRF-1: *P* < 0.05 and PGC-1*α* and TFAM: *P* < 0.01) and upregulated in the WT-D-gal-PTX group compared with the WT-D-gal group (mRNA levels: *PGC-1α*: *P* < 0.05 and *NRF-1* and *TFAM*: *P* < 0.01; protein levels: PGC-1*α*: *P* < 0.01 and NRF-1 and TFAM: *P* < 0.05). PTX administration increased the expression of PGC-1*α*, NRF-1, and TFAM in the brains of D-gal-induced aging mice, which indicated that the PTX treatment promoted mitochondrial biogenesis upon aging.

### 3.6. Effects of PTX Administration on Oxidative Balance Status and Mitochondrial Biogenesis in D-Gal-Induced Aging Nrf2-Deficient Mouse Brains

**Figure 5:**
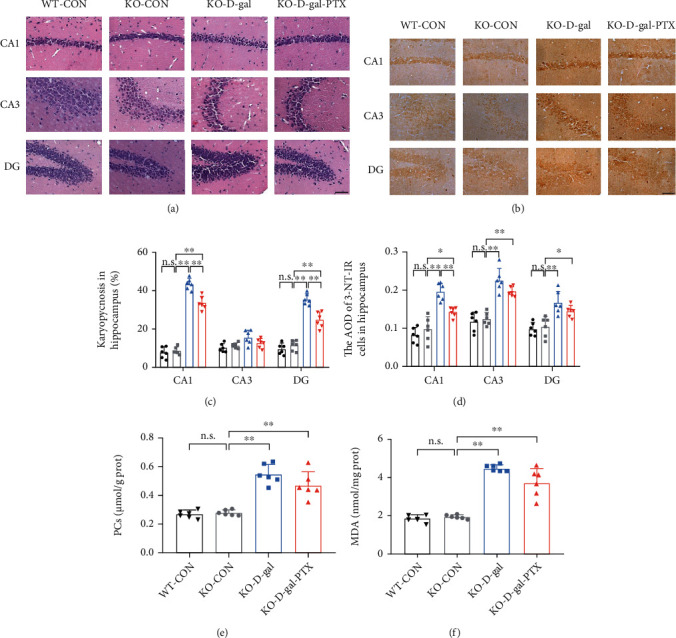
Effects of PTX administration on oxidative balance status in the hippocampus of D-gal-induced aging Nrf2-deficient mice as determined by (a, c) HE staining for karyopycnosis, (b, d) IHC staining for 3-NT, (e) PC assay, and (f) MDA detection. Scale bars = 50 *μ*m. Data are expressed as the mean ± S.D. (*n* = 6 mice/group). ^∗^*P* < 0.05 and ^∗∗^*P* < 0.01.

According to the crucial function of Nrf2 in PTX-treated aging mice, we next detected alterations in the oxidative balance status and mitochondrial biogenesis in D-gal-induced aging mouse brains following Nrf2 deficiency. The percentage of karyopycnosis (Figures 5(a) and 5(c), CA1 and DG regions, *P* < 0.01), the AOD of 3-NT-IR cells (Figures 5(b) and 5(d), *P* < 0.01), and the levels of PCs (Figures 5(e), *P* < 0.01) and MDA (Figure 5(f), *P* < 0.01) in the hippocampus significantly differed among the WT-CON, KO-CON, KO-D-gal, and KO-D-gal-PTX groups. Significant differences were not observed between the WT-CON and KO-CON groups in the above four parameters, indicating that Nrf2 deficiency has little effect on the oxidative balance status without stress. Post hoc tests revealed that the percentage of karyopycnosis (except for the CA3 region), the AOD of 3-NT-IR cells, and the levels of PCs and MDA were significantly elevated in the KO-D-gal group compared to the KO-CON group (*P* < 0.01). In addition, the karyopycnosis in the CA1 and DG regions and the 3-NT-IR AOD in the CA1 region were reduced in the KO-D-gal-PTX group, but did not reach normal levels (*P* < 0.01). The PCs and MDA levels showed a slight reduction in the KO-D-gal-PTX group, but the difference was not significant.

**Figure 6:**
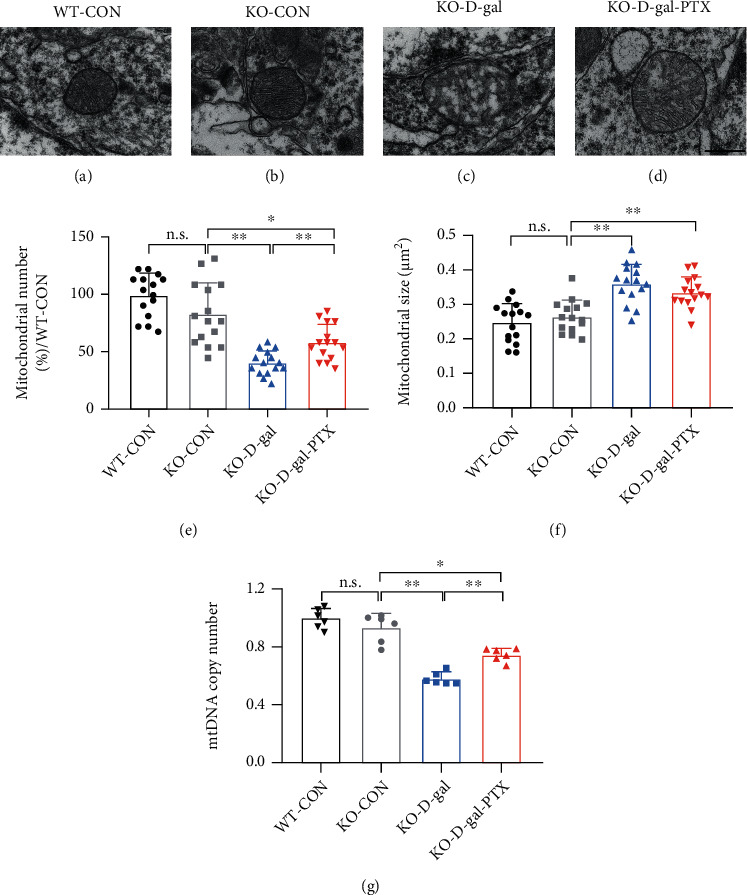
Effects of PTX administration on mitochondrial biogenesis in the hippocampus of D-gal-induced aging Nrf2-deficient mice. Using EM, mitochondrial ultrastructure images were collected from the (a) WT-CON, (b) KO-CON, (c) KO-D-gal, and (d) KO-D-gal-PTX groups. Scale bars = 500 nm. Quantitative morphometric measurements of the average level of (e) mitochondrial number and (f) mitochondrial size based on an analysis of 5 images per sample from each group (*n* = 3 mice/group). (g) mtDNA copy number was tested to measure mitochondrial content among the four groups (*n* = 6 mice/group). Data are expressed as the mean ± S.D.^∗^*P* < 0.05 and ^∗∗^*P* < 0.01.

The EM results showed that the mitochondrial ultrastructure in the WT-CON and KO-CON groups was normal (Figures 6(a) and 6(b)). However, the mitochondria from the KO-D-gal group showed massive swelling with architectural disruption (Figure 6(c)), which was consistent with the WT-D-gal group. The above abnormal ultrastructural alterations in mitochondria were ameliorated by the PTX treatment to a certain extent (Figure 6(d)). Furthermore, the mitochondrial number and size as well as the mtDNA copy number significantly differed among the experimental groups (Figures 6(e)–6(g), *P* < 0.01). A reduction in mitochondrial number and mtDNA copy number and an increase in mitochondrial size were detected in the KO-D-gal group compared with the KO-CON group (*P* < 0.01). After PTX administration, the mitochondrial number and mtDNA copy number were enhanced but did not reach normal levels (*P* < 0.01), while differences were not observed in mitochondrial size.

### 3.7. Effects of PTX Administration on Antioxidant and Mitochondrial Biogenesis-Related Gene Expression in D-Gal-Induced Aging Nrf2-Deficient Mouse Brains

**Figure 7:**
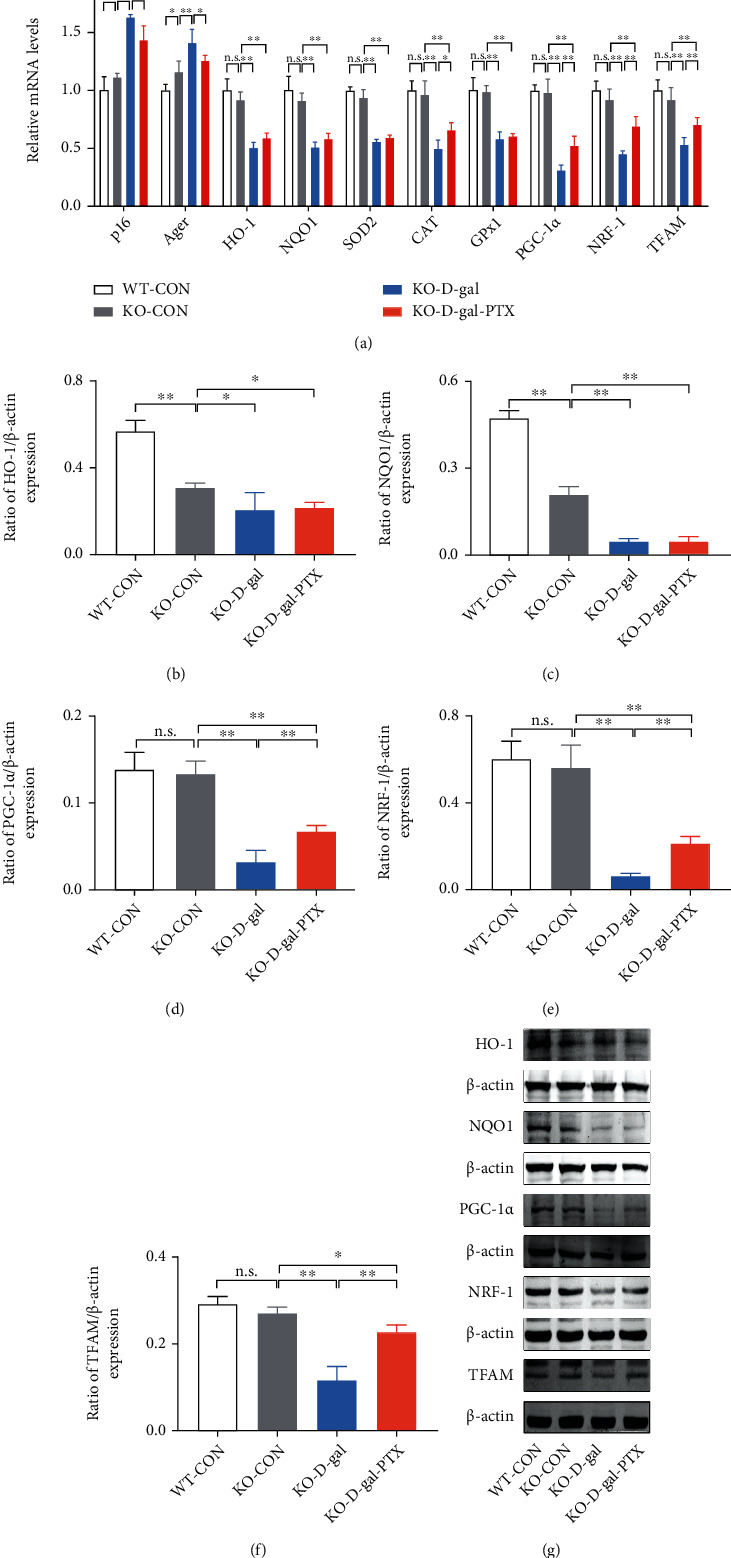
Effects of PTX administration on the expression of antioxidant and mitochondrial biogenesis-related genes in D-gal-induced aging Nrf2-deficient mouse brains. *p16*, *Ager*, *HO-1*, *NQO1*, *SOD2*, *CAT*, *GPx1*, *PGC-1α*, *NRF-1*, and *TFAM* mRNA levels were detected by qPCR. (a) *GAPDH* was used as an internal control. Densitometry analysis of (b) HO-1/*β*-actin, (c) NQO1/*β*-actin, (d) PGC-1*α*/*β*-actin, (e) NRF-1/*β*-actin, and (f) TFAM/*β*-actin. (g) Representative Western blots of the related protein levels mentioned above. Data are expressed as the mean ± S.D. (*n* = 6 mice/group). ^∗^*P* < 0.05 and ^∗∗^*P* < 0.01.

Based on changes in the oxidative balance status and mitochondria in D-gal-induced aging Nrf2-deficient mice, we next analyzed the expression of senescence-, antioxidant-, and mitochondrial biogenesis-related genes in the hippocampus. Group differences in *p16*, *Ager*, *HO-1*, *NQO1*, *SOD2*, *CAT*, *GPx1*, *PGC-1α*, *NRF-1*, and *TFAM* mRNA levels (Figure 7(a), *P* < 0.01) as well as HO-1, NQO1, PGC-1*α*, NRF-1, and TFAM protein levels (Figures 7(b)–7(g), *P* < 0.01) were found among the WT-CON, KO-CON, KO-D-gal, and KO-D-gal-PTX groups. As shown in Figure 7, significant differences were not observed between the WT-CON and KO-CON groups in *p16*, *HO-1*, *NQO1*, *SOD2*, *CAT*, *GPx1*, *PGC-1α*, *NRF-1*, and *TFAM* mRNA levels or PGC-1*α*, NRF-1, and TFAM protein levels. HO-1 and NQO1 protein levels were reduced in the KO-CON group following Nrf2 deficiency (*P* < 0.01). The results in the KO-D-gal group indicated that the D-gal treatment upregulated *p16* and *Ager* mRNA levels (*P* < 0.01), downregulated *HO-1*, *NQO1*, *SOD2*, *CAT*, and *GPx1* mRNA levels (*P* < 0.01), and reduced HO-1, NQO1, PGC-1*α*, NRF-1, and TFAM protein levels (HO-1: *P* < 0.05; others: *P* < 0.01) in the hippocampus compared to the KO-CON group. However, the mRNA expression of *HO-1*, *NQO1*, *SOD2*, *CAT*, and *GPx1* and the protein expression of HO-1 and NQO1 were elevated to a small extent in the KO-D-gal-PTX group compared to the KO-D-gal group (*CAT* mRNA levels: *P* < 0.05; others: not significant). The *p16* and *Ager* mRNA levels were reduced (*P* < 0.05), and the PGC-1*α*, NRF-1, and TFAM mRNA and protein levels were elevated (*P* < 0.01) in the KO-D-gal-PTX group compared to the KO-D-gal group. These findings were consistent with the morphological changes in hippocampal neurons and alterations in mitochondrial content. The results suggested that PTX administration still mildly attenuated the D-gal-induced aging and impairments of mitochondrial biogenesis but slightly enhanced the antioxidative capability in D-gal-induced aging mouse brains with Nrf2 deficiency. Therefore, in addition to the Nrf2-ARE pathway, additional molecular mechanisms may be involved in the response to PTX-induced mitochondrial biogenesis and antiaging effects.

### 3.8. Effects of PTX Administration on H_2_O_2_-Induced Changes in CREB, Nrf2, HO-1, and PGC-1*α* Expression Depend on CREB Pathway Activation

**Figure 8:**
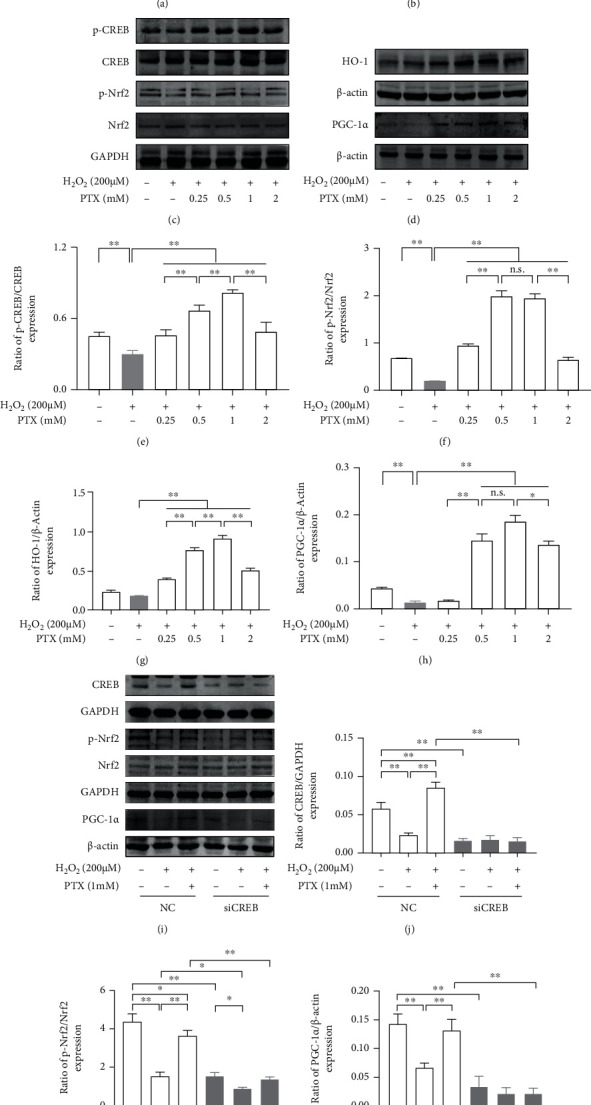
Effects of PTX administration on H_2_O_2_-induced changes in CREB, Nrf2, HO-1, and PGC-1*α* expression depend on CREB pathway activation. (a) Cell viability assessment in SH-SY5Y cells treated with different concentrations of PTX for 2 h to determine the maximum nontoxic dose by MTT assay. (b) Cell viability assessment in H_2_O_2_-induced SH-SY5Y cells treated with PTX. (c, d) Representative Western blots of p-CREB, CREB, p-Nrf2, Nrf2, HO-1, and PGC-1*α* protein levels in H_2_O_2_-induced SH-SY5Y cells treated with PTX. Densitometry analysis of (e) p-CREB/CREB, (f) p-Nrf2/Nrf2, (g) HO-1/*β*-actin, and (h) PGC-1*α*/*β*-actin. (i) Representative Western blots of CREB, p-Nrf2, Nrf2, and PGC-1*α* protein levels in CREB-silenced and H_2_O_2_-induced SH-SY5Y cells treated with PTX. Densitometry analysis of (j) CREB/GAPDH, (k) p-Nrf2/Nrf2, and (l) PGC-1*α*/*β*-actin. NC: siRNA-negative control. Data are expressed as the mean ± S.D. (*n* = 3/group). ^∗^*P* < 0.05 and ^∗∗^*P* < 0.01.

According to the results of the PTX treatment in D-gal-induced aging Nrf2-deficient mice, a typical oxidative stress injury model induced by H_2_O_2_ was selected and established to test and verify the effects of PTX on the CREB and Nrf2/PGC-1*α* signaling pathways. The H_2_O_2_ treatment dose-dependently decreased the viability of SH-SY5Y cells, and the cell viability was lowered by 46.73 ± 4.04% in the 200 *μ*M H_2_O_2_ treatment (Figure S1 (a)). Thus, 200 *μ*M H_2_O_2_ was used in the following experiments. We first evaluated the maximum nontoxic dose of PTX by measuring cell viability (Figure 8(a), *P* < 0.01). The results indicated that the maximum nontoxic dose of PTX was lower than 2 mM (*P* < 0.01).

To explore the effects of PTX on H_2_O_2_-induced changes in the CREB and Nrf2/PGC-1*α* signaling pathways, group differences in cell viability, as well as the protein expression of p-CREB, p-Nrf2, HO-1, and PGC-1*α*, were analyzed among the control, H_2_O_2_, and four PTX pretreatment groups (0.25 mM PTX+H_2_O_2_, 0.5 mM PTX+H_2_O_2_, 1 mM PTX+H_2_O_2_, and 2 mM PTX+H_2_O_2_) (Figures 8(b)–8(h), *P* < 0.01). Cell viability was increased in the four PTX pretreatment groups compared to the H_2_O_2_ group (*P* < 0.01). Among the four PTX pretreatment groups, cell viability in the 1 mM PTX+H_2_O_2_ group was the highest (relative to 0.25 mM PTX: *P* < 0.01 and relative to 2 mM PTX: *P* < 0.05). In addition, we found that the H_2_O_2_ treatment downregulated p-CREB, p-Nrf2, and PGC-1*α* protein levels compared to the control group (*P* < 0.01) while the four PTX pretreatment groups showed upregulated p-CREB, p-Nrf2, and HO-1 levels compared to the H_2_O_2_ group (*P* < 0.01). In addition, except for the 0.25 mM PTX+H_2_O_2_ group, PGC-1*α* protein levels were elevated in the other three PTX pretreatment groups relative to the H_2_O_2_ group (*P* < 0.01). Moreover, p-CREB and HO-1 protein levels were increased in the 1 mM PTX pretreatment group compared to the 0.5 mM or 2 mM PTX pretreatment group (*P* < 0.01). The p-Nrf2 and PGC-1*α* protein levels were increased in the 0.5 mM and 1 mM PTX pretreatment group compared to the 0.25 mM or 2 mM PTX pretreatment group (p-Nrf2: *P* < 0.01 and PGC-1*α*: *P* < 0.05). In summary, the 1 mM PTX pretreatment more effectively elevated the expression of p-CREB, p-Nrf2, HO-1, and PGC-1*α* in H_2_O_2_-induced SH-SY5Y cells.

Finally, we silenced the CREB gene in SH-SY5Y cells by CREB siRNA transfection to investigate the role of CREB in PTX-induced effects on antioxidant and mitochondrial biogenesis. The CREB mRNA and protein levels significantly differed among the NC, siCREB-1, siCREB-2, and siCREB-3 groups (Figure S1 (b-d), *P* < 0.01). The mRNA and protein expression levels in the siCREB-1 group were markedly reduced by 70.45% and 55.49%, compared with those in the NC group, respectively (*P* < 0.01). According to the siRNA silencing efficiency, siCREB-1 was used in the following experiments. Group differences in CREB, p-Nrf2, and PGC-1*α* protein expression were observed among the NC, NC-H_2_O_2_, NC-H_2_O_2_-PTX, siCREB, siCREB-H_2_O_2_, and siCREB-H_2_O_2_-PTX groups (Figures 8(i)–8(l), *P* < 0.01). Compared with the NC group, the CREB, p-Nrf2, and PGC-1*α* protein levels in the NC-H_2_O_2_ group were reduced by 59.65%, 64.28%, and 52.86%, respectively (*P* < 0.01); however, when the CREB gene was silenced, these three protein levels were decreased by 72.77%, 64.95%, and 76.28%, respectively (*P* < 0.01). CREB, p-Nrf2, and PGC-1*α* protein levels were elevated in the NC-H_2_O_2_-PTX group compared to the NC-H_2_O_2_ group (*P* < 0.01), which was consistent with the preceding findings. Except for a reduction in p-Nrf2 expression in the siCREB-H_2_O_2_ group relative to the siCREB group (*P* < 0.05), no significant differences were found among the siCREB, siCREB-H_2_O_2_, and siCREB-H_2_O_2_-PTX groups in the three protein expression levels. These data suggested that CREB and Nrf2/PGC-1*α* act in a linear way, and the beneficial effects of PTX administration on H_2_O_2_-induced changes might depend on CREB pathway activation.

## 4. Discussion

In the present study, we investigated the effects and mechanisms of PTX administration on the aging process. PTX treatment significantly ameliorated the cognitive deficits, enhanced the antioxidative capability, and promoted mitochondrial biogenesis in D-gal-induced aging wild-type mice, although these antiaging effects were partially attenuated in the Nrf2-deficient aging mice. In addition, we found that positive feedback may occur between the CREB and Nrf2/PGC-1*α* pathways in a CREB gene-silenced cell model treated with H_2_O_2_. Hence, PTX-induced, Nrf2-, and PGC-1*α*-dependent increases in antioxidative capability and mitochondrial biogenesis might depend on CREB pathway activation, which underlies the antiaging effects of PTX.

Systemic administration of D-gal induced cognitive deficits, aggravated oxidative damage (increased karyopycnosis, AOD of 3-NT-IR, PC content, and MDA levels), destroyed the mitochondrial structure, reduced the mitochondrial content (decreased mitochondrial number, CS activity, and mtDNA copy number), decreased Nrf2 activation, upregulated senescence-associated (*p16* and *Ager*) mRNA levels, and downregulated antioxidant (HO-1, NQO1, SOD2, CAT, and GPx1)-related and mitochondrial biogenesis (PGC-1*α*, NRF-1, and TFAM)-related gene expression. All of the changes suggested that the aging mouse model was successfully established by daily subcutaneous injection of 100 mg/kg D-gal for 8 weeks. The results in the wild-type D-gal-induced aging model demonstrated that PTX facilitated cognitive capability, protected hippocampal neuronal cells against D-gal-induced oxidative damage, and prompted mitochondrial biogenesis in the aging mouse brain. We found that the increases in antioxidative capability and mitochondrial biogenesis might form the basis for the antiaging effects of PTX and that the activation of Nrf2 might be responsible for the mechanisms.

Nrf2 is a member of the Cap'n'collar transcription factor family and plays a central role in initiating the expression of a majority of endogenous antioxidant enzymes [47, 48]. Nrf2 is sequestered by Keap1- (Kelch-like ECH-associated protein 1-) based E3 ligase complexes in the cytosol and rapidly degraded by ubiquitin. Upon exposure to stress, Nrf2 is isolated from Keap1, phosphorylated by protein kinase, and then translocated to the nucleus. Once in the nucleus, Nrf2 forms a heterodimer with small Maf proteins and binds to ARE, which promotes the expression of proteins involved in the response to redox homeostasis [49, 50]. In addition, activated Nrf2 directly enhances PGC-1*α* expression [14, 17], and PGC-1*α* or activated Nrf2 regulates NRF-1 expression by binding to the NRF-1 promoter region [51, 52]. NRF-1 further activates TFAM, which is directly involved in mtDNA transcription and replication, thus promoting mitochondrial biogenesis [31, 53].

Nrf2-deficient mice of a young age display no overt phenotypic differences relative to wild-type mice but are extremely vulnerable to various toxic insults, which is consistent with the results in this study [54, 55]. Administration of PTX partially alleviated the oxidative damage, slightly improved antioxidative capability, and mildly exerted protective effects on mitochondrial biogenesis against D-gal-induced damage following Nrf2 deficiency. These findings indicated that the enhanced activation of Nrf2 induced by PTX played an important role in resisting oxidative damage during aging. Moreover, PTX-induced mitochondrial biogenesis was partially regulated by Nrf2 activation. Nrf2 and PGC-1*α* possibly form a feedback loop with each other to regulate the expression of antioxidant and mitochondrial biogenesis genes [56–58]. Previous studies revealed that PGC-1*α* positively regulates the activation of Nrf2 via the inhibition of glycogen synthase kinase 3*β* (GSK3*β*). GSK3*β* is deactivated by p38, which is activated by PGC-1*α* [59]. Thus, another molecular mechanism must occur between PTX and the PGC-1*α* pathway to promote mitochondrial biogenesis.

The main pharmacological activities of PDE inhibitor can be explained by inhibition of PDEs, which is responsible for the breakdown of the intracellular second messengers, cAMP or cGMP. Consistent with the effects of PTX, several synthetic or natural molecules inhibiting various PDE subtypes such as cilostazol, milrinone, rolipram, sildenafil, tadalafil, BAY 73-6691, and caffeine have been reported showing encouraging results for the treatment of neurodegenerative disorders, which might be closely related to increase mitochondrial biogenesis and antioxidant activities [60–66]. Our previous research on the antiaging effects of PTX suggested that PTX administration increased the cAMP content in aged rats by preventing the inactivation of cAMP [22]. The elevated cAMP content activates cAMP-dependent protein kinase A (PKA), thus leading to an increased level of the phosphorylation of CREB on Ser133 by recruitment of the coactivator CREB-binding protein (CBP) [27, 29, 67]. Moreover, evidence has been obtained that cross-talk occurs between the Nrf2 and CREB pathways by binding to CBP, which leads to their cooperation in the expression of Nrf2 target genes, such as HO-1, NQO1, and SOD2 [29, 32, 68, 69]. Additionally, PGC-1*α* has been shown to be directly regulated by p-CREB [9, 30, 31]. In this study, administration of PTX in H_2_O_2_-induced SH-SY5Y cells led to increases in CREB and Nrf2 phosphorylation as well as upregulation of HO-1 and PGC-1*α* protein levels. Furthermore, siRNA-mediated silencing of CREB abrogated the PTX-induced upregulation of p-Nrf2 and PGC-1*α*, which suggested that CREB and Nrf2/PGC-1*α* act in a linear pathway.

Although the results suggested that a strong link occurred between PTX-induced antiaging effects and CREB and Nrf2/PGC-1*α* pathway activation, some limitations of the current study should be considered when interpreting these results. First, we only concentrated on the effects of both D-gal and PTX in neurons, but did not examine the potential effects on astrocytes and microglia. In addition, some indices need to be evaluated after PTX administration, such as a screening of the mediators to transduce the signaling of PTX, evaluation of the blood circulation, and turnover of the feedstuff for mitochondrial biogenesis, which will provide further evidence for antiaging mechanisms of PTX. Moreover, because PDEs inhibited by PTX are distributed in many organs of the organism, the oxidative balance status and mitochondrial biogenesis of different tissues should be tested to further elucidate the effects of PTX on different organs. Furthermore, the relationships among antioxidants, mitochondrial biogenesis, and other antiaging pathways are complex and closely related. Hence, further studies are required to understand the precise molecular mechanisms of PTX-induced effects on the aging brain.

**Figure 9:**
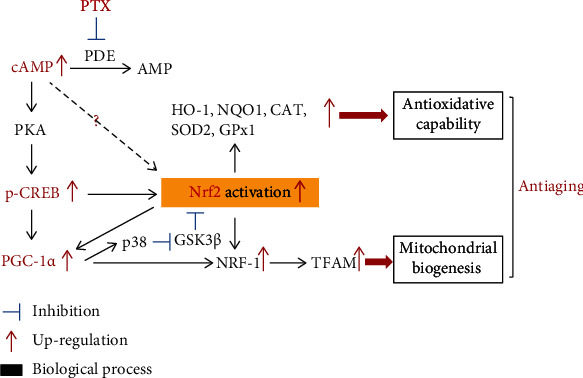
Possible molecular mechanisms of PTX-induced antiaging effects.

In conclusion, the detrimental effects of aging in the brain could be prevented by PTX treatment, which ameliorated cognitive deficits; decreased oxidative damage to proteins, nucleic acids, and lipids; improved the mitochondrial ultrastructure; and increased mitochondrial contents in aging mouse brains. Thus, PTX administration effectively improved the aging-related decline in brain function by enhancing antioxidative capability and promoting mitochondrial biogenesis, and these processes might depend on increasing Nrf2 and PGC-1*α* through the cAMP-CREB pathway (Figure 9). Together, these findings provide insights into the potential applications and molecular mechanisms of PTX in delaying the aging process.

## Figures and Tables

**Table 1:** qPCR primer sequences.

Gene	Forward primer (5′-3′)	Reverse primer (5′-3′)
For mouse genes		
Senescence		
*p16*	GAACTCTTTCGGTCGTACCC	CGAATCTGCACCGTAGTTGA
*Ager*	ACTACCGAGTCCGAGTCTACC	CCCACCTTATTAGGGACACTGG
Oxidative stress		
*HO-1*	CAACGAATCTTGAATGGAGG	AGGTGTCCAGAGAAGGCTT
*NQO1*	ATCCTTCCGAGTCATCTCTA	CAACGAATCTTGAATGGAGG
*SOD2*	TGAACAACCTCAACGCCAC	GAAGGTAGTAAGCGTGCTC
*CAT*	AACTGGGATCTTGTGGGAA	GACAGTTCACAGGTATCTG
*GPx1*	CTCACCCGCTCTTTACCTTCCT	ACACCGGAGACCAAATGATGTACT
Mitochondrial biogenesis		
*PGC-1α*	GAAAGGGCCAAACAGAGAGA	GTAAATCACACGGCGCTCTT
*NRF-1*	TGGAGTCCAAGATGCTAATG	AGAGCTCCATGCTACTGTTC
*TFAM*	CAGGAGGCA AAGGATGATTC	CCA AGACTTCATTTCATTGTCG
Housekeeping gene		
*GAPDH*	ACTCTTCCACCTTCGATGCC	TCTTGCTCAGTGTCCTTGCT
For human genes		
*CREB*	ATCTTAGTGCCCAGCAACCA	ACATGTTACCATCTTCAAACTGACG
*GAPDH*	AGAAGGCTGGGGCTCATTTG	AGGGGCCATCCACAGTCTTC

## Data Availability

The data used to support the findings of this study are included within the article.

## Supplementary Materials

Supplementary Materials Figure S1: cell viability assessment in SH-SY5Y cells treated with different concentrations of H_2_O_2_ (0, 50, 100, 200, 400, or 800 *μ*M) for 24 h to determine the appropriate dose by MTT assay (a). *CREB* mRNA levels among the NC, siCREB-1, siCREB-2, and siCREB-3 groups were detected by qPCR. *GAPDH* was used as an internal control (b). Representative Western blots of CREB protein levels (c). Densitometry analysis of CREB/GAPDH (d). Data are expressed as the mean ± S.D. (*n* = 3/group). ^∗^*P* < 0.01. Table S1: accession numbers of the genes for primers.
