# Supplementary material for: Pentoxifylline Enhances Antioxidative Capability and Promotes Mitochondrial Biogenesis in D-Galactose-Induced Aging Mice by Increasing Nrf2 and PGC-1α through the cAMP-CREB Pathway
Source: Oxid Med Cell Longev. 2021 Jun 22;2021:6695613. doi: 10.1155/2021/6695613 (PMC8245236; doi:10.1155/2021/6695613)
Supplement: Supplementary Materials — Figure S1: cell viability assessment in SH-SY5Y cells treated with different concentrations of H2O2 (0, 50, 100, 200, 400, or 800 μM) for 24 h to determine the appropriate dose by MTT assay (a). CREB mRNA levels among the NC, siCREB-1, siCREB-2, and siCREB-3 groups were detected by qPCR. GAPDH was used as an internal control (b). Representative Western blots of CREB protein levels (c). Densitometry analysis of CREB/GAPDH (d). Data are expressed as the mean ± S.D. (n = 3/group). ∗P < 0.01. Table S1: accession numbers of the genes for primers. [file 6695613.f1.zip › 6695613.f1/Table S1.docx]

TABLE S1: Accession numbers of the genes for primers.

| Gene | Accession numbers |
| --- | --- |
| *p16* | XM_021160984.1 |
| *Ager* | XM_021186427.2 |
| *Nrf2* | XM_021193142.2 |
| *HO-1* | U12961.1 |
| *NQO1* | XM_029480809.1 |
| *SOD2* | X04972.1 |
| *CAT* | XM_021194769.2 |
| *GPx1* | XM_021207183.2 |
| *PGC-1α* | XM_029477627.1 |
| *NRF-1* | AF098077.1 |
| *TFAM* | XM_029542576.1 |
| *GAPDH* | BC110311.1 |
| *HK2 (nDNA)* | JN957201.1 |
| *16S rRNA (mtDNA)* | LC062083.1 |
| *CREB (human)* | NM_001320793.2 |
| *GAPDH (human)* | M17851.1 |
