# Supplementary figures and images for: Pentoxifylline Enhances Antioxidative Capability and Promotes Mitochondrial Biogenesis in D-Galactose-Induced Aging Mice by Increasing Nrf2 and PGC-1α through the cAMP-CREB Pathway
Source: Oxid Med Cell Longev. 2021 Jun 22;2021:6695613. doi: 10.1155/2021/6695613 (PMC8245236; doi:10.1155/2021/6695613)

## Slide 1
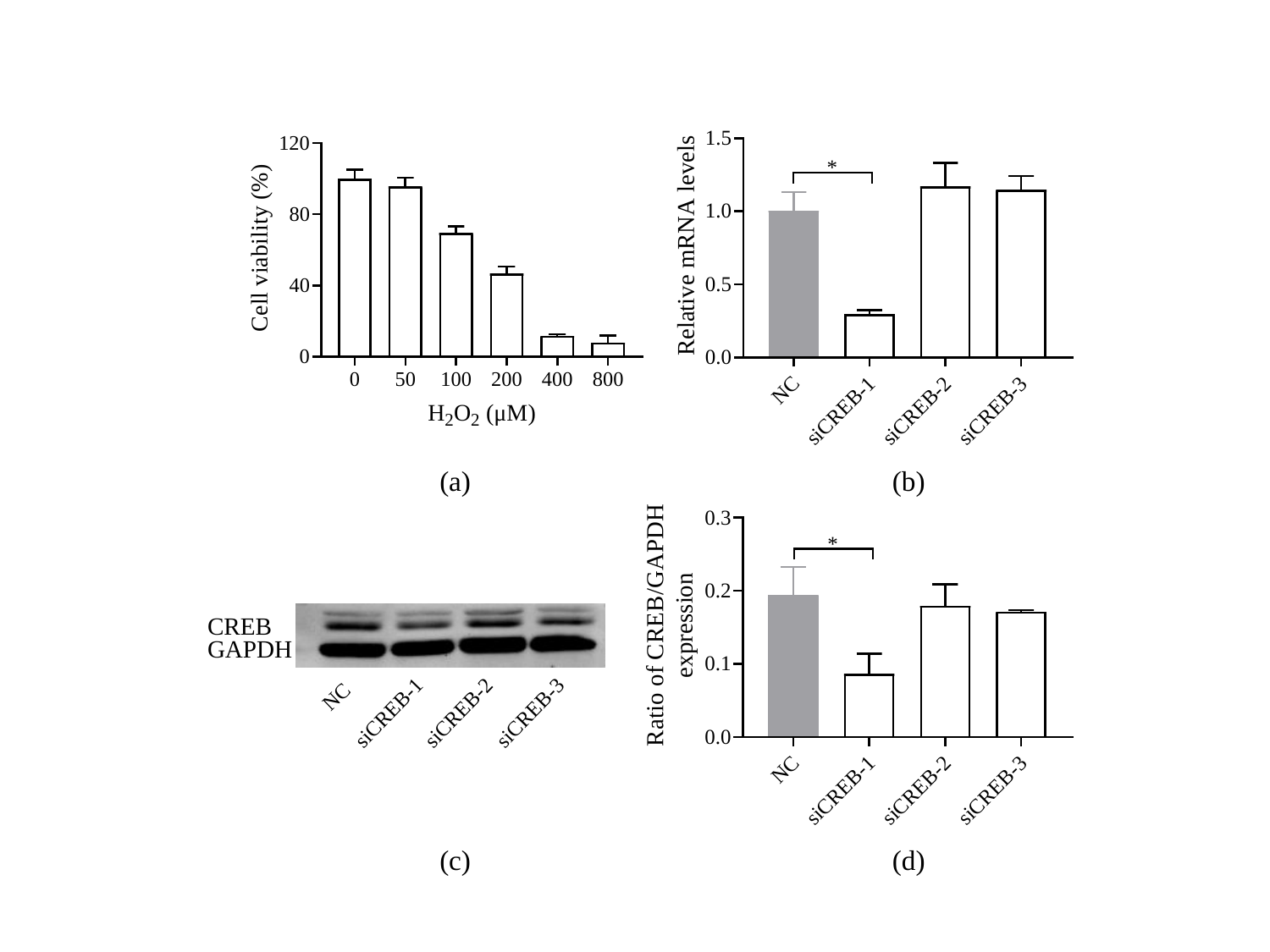

(a)
(b)
CREB
GAPDH
NC
siCREB-1
siCREB-2
siCREB-3
(c)
(d)

Supplement: Supplementary Materials — Figure S1: cell viability assessment in SH-SY5Y cells treated with different concentrations of H2O2 (0, 50, 100, 200, 400, or 800 μM) for 24 h to determine the appropriate dose by MTT assay (a). CREB mRNA levels among the NC, siCREB-1, siCREB-2, and siCREB-3 groups were detected by qPCR. GAPDH was used as an internal control (b). Representative Western blots of CREB protein levels (c). Densitometry analysis of CREB/GAPDH (d). Data are expressed as the mean ± S.D. (n = 3/group). ∗P < 0.01. Table S1: accession numbers of the genes for primers. [file 6695613.f1.zip › 6695613.f1/Figure S1.pptx]
